# Dispersal of Biofilms by Secreted, Matrix Degrading, Bacterial DNase

**DOI:** 10.1371/journal.pone.0015668

**Published:** 2010-12-14

**Authors:** Reindert Nijland, Michael J. Hall, J. Grant Burgess

**Affiliations:** 1 Dove Marine Laboratory, School of Marine Science and Technology, Newcastle University, North Shields, United Kingdom; 2 School of Chemistry, Newcastle University, Newcastle upon Tyne, United Kingdom; Auburn University, United States of America

## Abstract

Microbial biofilms are composed of a hydrated matrix of biopolymers including polypeptides, polysaccharides and nucleic acids and act as a protective barrier and microenvironment for the inhabiting microbes. While studying marine biofilms, we observed that supernatant produced by a marine isolate of *Bacillus licheniformis* was capable of dispersing bacterial biofilms. We investigated the source of this activity and identified the active compound as an extracellular DNase (NucB). We have shown that this enzyme rapidly breaks up the biofilms of both Gram-positive and Gram-negative bacteria. We demonstrate that bacteria can use secreted nucleases as an elegant strategy to disperse established biofilms and to prevent *de novo* formation of biofilms of competitors. DNA therefore plays an important dynamic role as a reversible structural adhesin within the biofilm.

## Introduction

In their natural environment most bacteria grow within surface attached communities known as biofilms. Bacterial biofilms are problematic in industrial settings, where they contribute to biofouling [1] and in human health, where they contribute directly to antibiotic resistant infections [2], [3]. Biofilms consist of sessile bacteria embedded within a hydrated extracellular matrix, with a physiology, gene expression pattern and morphology that is distinct from planktonic cells [4], [5], [6].

The extracellular matrix contains a complex arrangement of extracellular polysaccharides and proteins as well as considerable quantities of extracellular DNA or eDNA [7]. The first observation of eDNA as a structural component in biofilms was by Catlin in 1956 [8] where he demonstrated not only that DNA could be isolated from the matrix itself but, in an elegant experiment, showed that the addition of bovine DNaseI significantly reduced the viscosity of bacterial biofilms, ultimately leading to dispersal. This work was further developed by Whitchurch [9] who showed that DNA is involved in the initial steps of adhesion and biofilm formation, and that bovine DNaseI inhibits biofilm formation for up to 60 hours after the biofilm growth is initiated. This has led to the use of both commercial bovine and recombinant human DNaseI in the disruption of medically important biofilms [10]. Treatment of antibiotic resistant biofilms with DNaseI has been shown to increase matrix permeability, resulting in a subsequent increase in antibiotic susceptibility [11].

Bacteria are capable of modifying the structure of their own biofilms, as part of their lifecycle. This can be carried out by the secretion of matrix degrading enzymes such as proteases and polysaccharide degrading enzymes such as amylases or Dispersin B [7], [12]. Furthermore, it has been recently shown that unusually dense biofilms are produced by a *Staphylococcus aureus* mutant which can no longer secrete its main thermonuclease [13]. Here we demonstrate for the first time that secreted bacterial nucleases can also be employed to control the development and dispersal of bacterial biofilms, presumably by degradation of structurally important nucleic acids.

## Materials and Methods

### Bacterial strains, media, growth conditions

Bacterial strains and plasmids used in this study are listed in Table 1. All strains were grown at 37°C under vigorous agitation in LB medium (VWR, UK) unless specified otherwise.

**Table 1:** Strains and Plasmids used.

Strains	Genotype	Source/Reference
*B. licheniformis* EI-34-6	Environmental isolate	[15]
*B. licheniformis* DSM13	Sequenced type strain	http://www.bgsc.org/
*B. subtilis* NZ8900	*trpC2, amyE::spaRK*; Km^R^	[17]
*B. subtilis* ATCC6633	Subtilin producer	[30]
*E. coli* DH5alpha		Invitrogen

Plasmids	Characteristics and description	Reference
pNZ8901	Cm^R^, shuttle vector, SpaS promoter	[17]
pNZ8902	Ery^R^, shuttle vector, SpaS promoter	[17]
pNZ8901-nucB	Cm^R^, *B. licheniformis* EI-34-6 nucB cds	This work
pNZ8902-nucB	Ery^R^, *B. licheniformis* EI-34-6 nucB cds	This work
pNZ8901-Barnase	Cm^R^, *B. licheniformis* EI-34-6 barnase cds	This work
pNZ8901-Barnase-Barstar	Cm^R^, *B. licheniformis* EI-34-6 barnase and barstar containing cds.	This work

*Km^R^: Kanamycin resistance, CmR: Chloramphenicol resistance, Ery^R^: Erythromycin resistance. cds: Coding sequence.*

### Strain constructions and transformation

The cloning and transformation procedures were performed according to established techniques [14] and suppliers' manuals. Restriction enzymes, DNA polymerases, DNase I, RNase I, T4 DNA ligase were obtained from Fermentas Life Sciences (Vilnius, Lithuania) and used as specified by the suppliers. Deoxynucleotide primers for PCR were obtained from Invitrogen (UK), and Table 2 lists the sequences of primers used.

**Table 2:** Primers used in this study.

nucB-fw+BsteII	ATAGGTGACCGTCATGATCAAAAAATGGGCGGTTCATCTGC
nucB-rv+XbaI	ATCTCTAGATATTTGTTTTTCGCCTTTTATTG
Barnase-fw+BstEII	ATAGGTGACCTCCATGAAAAAAATATTATCAACTC
Barnase-rv+hindIII	CTAGAAGCTTCATATGATCATCTCATTCTCGTAAAC
barstar-RV_HindIII	GTAGAAGCTTGAAGCGCCCGCTCGTTTTCTGTT

(Restriction sites are underlined).

### Production of AMS supernatant from *Bacillus licheniformis* EI-34-6

*B. licheniformis* EI-34-6 was grown in 10 ml Air Membrane Surface (AMS) bioreactors as described previously [15] in NGF medium (Nutrient broth (Oxoid) 13 g/l, 1% glycerol, 1 mM FeCl_2_). After 7 days of growth, the medium underneath the filter membranes was collected, pooled, centrifuged at 7800 rpm in 50 ml falcon tubes for 10 min and filtered using a 0.2 µm syringe filter to ensure sterility.

### Biofilm formation inhibition and dispersal screening method

Biofilm dispersal was screened using clear 96 well flat bottom polystyrene tissue culture plates (BD-Falcon, USA). *Bacillus licheniformis* DSM13 and other bacterial strains tested were grown for 48–96 h and diluted 1∶100 in fresh LB. 200 µl of this culture was added to every well of a 96 well plate. To test for inhibition of biofilm formation, the biofilm dispersal compound was added directly, and the plate was incubated at 37°C, without shaking, for 20–28 h to allow for biofilm development. To test for dispersal activity, the biofilm dispersal compounds were added in varying concentrations after 20–28 h of growth and biofilm development at 37°C, and the plate was further incubated for 1 h at 37°C. Then all non-attached cells were removed by discarding the culture medium and rinsing the plate in a container by immersing and agitating gently four times in tap water. Attached biofilm material was stained by addition of 250 µl of 0.5% crystal violet solution (CV) to each well of the plate for 10 min. Unbound CV stain was removed by aspiration and the plate was rinsed again in tap water until no more CV was observed to dissolve in the water. The plates were air dried and photographed. Subsequently, 250 µl of 96% ethanol containing 2% acetic acid (v/v) was added to each well. Adsorption at 595 nm was measured using a Fluostar Optima plate reader (BMG Labtech, UK), and the data was analysed using the MARS software package (BMG Labtech, UK) and Microsoft Excel.

### Isolation and Bioassay guided fractionation of proteins from the supernatant

The proteins in the AMS supernatant were concentrated 50 fold by precipitation with trichloroacetic acid (TCA) (Sigma, UK) as follows: The supernatant of several AMS cultures was pooled, and 6.1 M TCA solution was added to give a final concentration of 0.9 M TCA. This solution was kept on ice for 30 minutes to allow for protein precipitation and the precipitated protein was collected through centrifugation (10 min at 7800 rpm in 50 ml falcon tubes). The protein containing pellets were washed twice using ice cold 96% ethanol, and air dried for 30 min at 45°C. Each pellet was dissolved in 1∶50^th^ of the original volume with 0.05 M Tris-HCl buffer (pH 7.0). This concentrate was fractionated using a Superose™ 12 (GE Healthcare, UK) gel filtration column (height 40 cm, diameter 3 cm) using ultra pure water as the mobile phase and fractions of 12 ml each were collected. The fractions were tested for biofilm dispersal activity using the 96 well microtitre plate setup, with crude supernatant as the positive control and H_2_O as the negative control. Proteins in the active fraction were concentrated again via TCA precipitation (as before) and analysed by SDS page.

### SDS-page and peptide mass fingerprinting

The single active fraction from gel filtration on Superose™ 12 was concentrated 10× via TCA precipitation in 2 ml micro-tubes and separated on a 4–12% Tris-Tricine gel using MES buffer (Invitrogen, UK). A Novex Sharp Pre-stained protein standard (Invitrogen, UK) was also loaded to determine protein size. After electrophoresis the gel was stained using Biosafe Coomassie (Biorad, UK) according to the manufacturer's protocol. Three bands were visible on the gel, one abundant band at 12 kDa and two higher bands around 30 and 34 kDa. These three bands were analysed by LCMS, following in-gel tryptic digest (North East Proteome Analysis Facility (www.nepaf.com), Newcastle, UK). The peptide fragments were analysed against NCBI NC_006270.faa 2008.04.22 (Bacillus_licheniformis_ATCC_14580), NCBI NC_006322.faa 2008.04.22 (Bacillus_licheniformis_DSM_13), NCBI C_000964.faa 2008.04.22 (Bacillus_subtilis) and the common Repository for Adventitious Proteins.

### Cloning and overexpression of NucB and Barnase in *Bacillus subtilis* NZ8900

Primers were designed to amplify both identified nuclease genes based on the published genome sequence of *B. licheniformis* DSM13 [16].

For barnase, primer sets were designed to amplify the gene only and also the barnase-barstar operon. PCR was performed using Phusion DNA polymerase (Finnzymes, Finland). Table 2 lists the nucleotide sequences of the primers used. The barnase gene and barnase-barstar operon were successfully amplified in one.

The PCR reaction to amplify *nucB* did result in several amplified fragments, and a faint band of the correct size was present. This band was isolated from the agarose gel (gel isolation kit, Invitrogen, UK) and used as a template for a new PCR. The amplified genes were digested with *Eco91I*, *XbaI* (*nucB*) and *Eco91I*, *HindIII* (*barnase*, *barnase-barstar*) and ligated into vectors pNZ8901 (Cm^R^) and pNZ8902 (Ery^R^). The ligation mixture was transformed to *E. coli* DH5 alpha. Colonies were screened using colony PCR with the unique primers mentioned above and plasmids were isolated from positive clones. Plasmids were analysed by restriction and correct plasmids were sequenced. Constructed plasmids are listed in Table 1. The constructed plasmids were transformed to *Bacillus subtilis* NZ8900 [17] using natural competence [18].

*B. subtilis* NZ8900+pNZ8901/2-nucB clones were screened on DNase test agar containing methyl Green (Oxoid, UK) as follows. A colony was streaked onto the DNase test agar and grown overnight at 30°C. Subsequently a drop of *B. subtilis* ATCC6633 culture supernatant containing subtilin and 1% agar was spotted next to the colony. The plates were further incubated for 2 h at 37°C and colonies developing a halo due to the degradation of DNA were judged positive. Correct *B. subtilis* clones containing the Barnase gene were characterized by colony-PCR followed by plasmid isolation and restriction analysis of the obtained plasmid.

Correct clones were picked from single colonies and transferred to a shake flask containing LB and the appropriate antibiotics (Kanamycin and Chloramphenicol/Erythromicin). At an OD_600_ of ∼1.0, 5% cell free supernatant of an overnight *B. subtilis* ATCC6633 culture was added to provide subtilin to induce expression and the total culture supernatant was harvested 2 h after induction.

Overproduction of the NucB or Barnase was visualised on SDS-page after 10× concentration via TCA precipitation. The concentration of overproduced NucB was estimated on SDS-page by comparing band intensity after staining the gel with Bio-Safe Coomassie (Biorad) against a BSA standard. When production of Barnase was induced the culture stopped growing and no overproduction of Barnase could be detected on a Coomassie stained PAA gel. To circumvent this problem the gene downstream of Barnase, barstar, was also included in the overexpression construct. This strategy yielded an improvement in Barnase overexpression.

### Testing of active fractions for DNase activity

DNase activity was tested by incubating purified plasmid DNA with the DNase containing fractions for 30 min at 37°C. The samples were run on a 1% agarose gel containing ethidium bromide to visualize DNA degradation.

## Results

**Figure 1:**
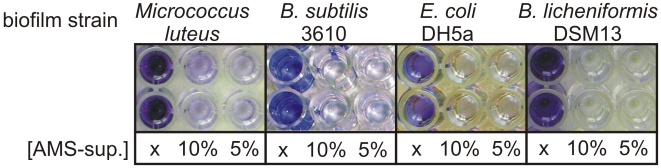
Dispersal of several bacterial species by AMS supernatant. Typical examples of dispersal of several 26 hour grown biofilm forming strains by AMS supernatant. Remaining biofilm visualised by CV staining after 30 minutes incubation with dispersal compound. x = control (only medium added), 10% = 10% of AMS supernatant added, 5% = 5% of AMS supernatant added.

**Figure 2:**
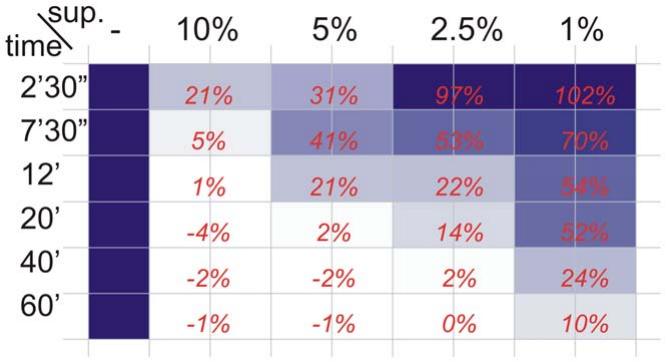
Dispersal efficiency in time and concentration. Efficiency of dispersal of *B. licheniformis* DSM13 24 hour old biofilm by AMS supernatant (sup.) visualised as remaining CV stain as measured by plate reader. Incubation time in minutes (') and seconds (”) indicated on the left, concentration of AMS supernatant indicated on top. The biofilm remaining is indicated with both a colour scale (dark blue: no dispersal, white: full dispersal) and as a percentage of non-dispersed biofilm (red numbers).

It has been shown previously that a marine isolate of *Bacillus licheniformis*, strain EI-34-6, produces the antibiotic bacitracin along side a red pigment when growing in an air-membrane surface (AMS) bioreactor, whereas this is not observed in standard planktonic growth in shakeflasks [13]. The supernatant from cultures grown in these biofilm conditions, but not during standard shakeflask growth, was observed to inhibit both biofilm formation and to disperse bacterial biofilms. Inhibition of formation and dispersal of the biofilms of both Gram-positive and Gram-negative bacteria including *B. licheniformis*, *B. subtilis, Escherichia coli*, *Micrococcus luteus* and *Pseudomonas* was observed in a standard biofilm dispersal assay using a 96 well plate format crystal violet staining, selected examples are shown in Fig. 1. Dispersal of existing biofilms was rapid and partial dispersal was visible within 2.5 min. At higher concentrations, dispersal was complete within 12 minutes (Fig. 2).

**Figure 3:**
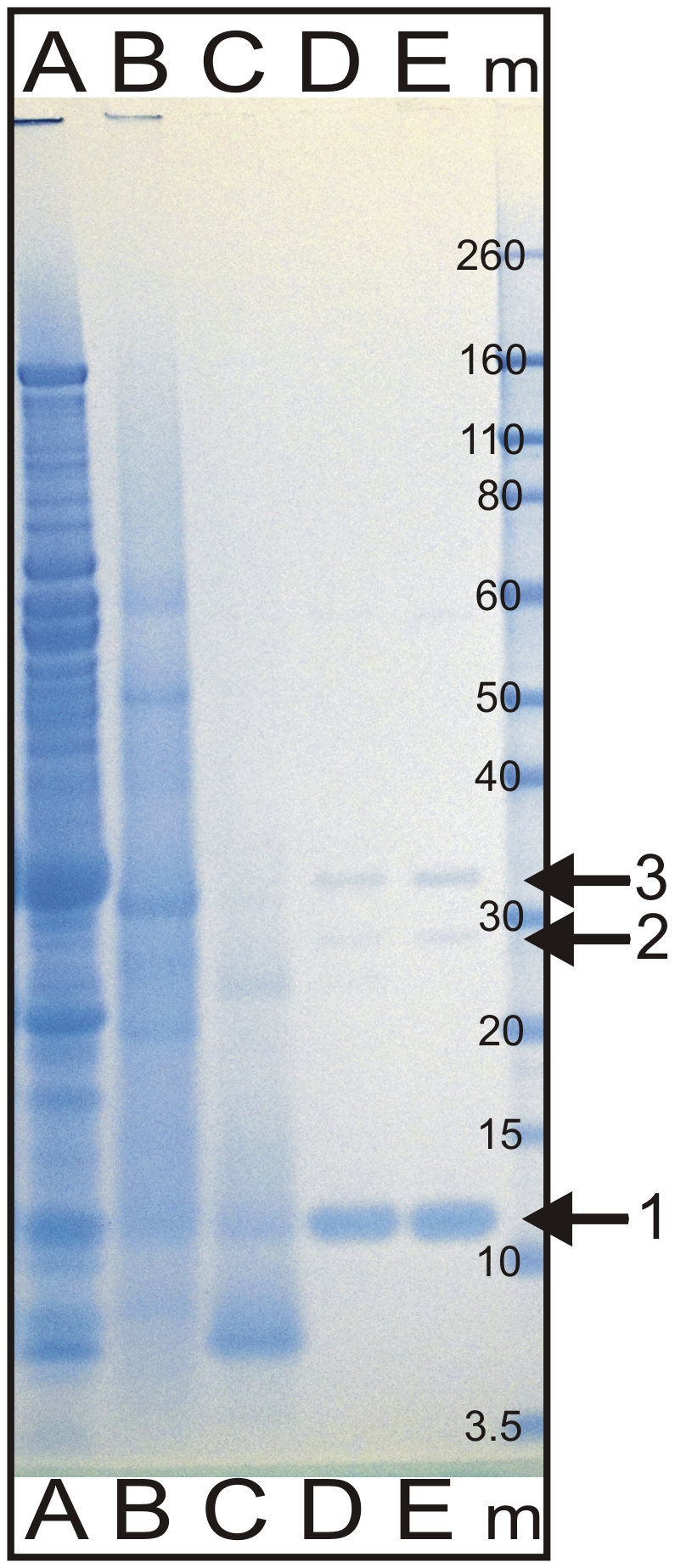
Efficiency of different AMS supernatant fractionation methods. **A**: total supernatant of the AMS culture; **B**: active fraction obtained after rotary evaporation followed by Sephadex G50 gel filtration; **C**: active fraction obtained after freeze-drying by Sephadex-LH20 gel filtration, **D+E**: Active fraction obtained by TCA precipitation followed by Superose 12 gel filtration. **m** = Invitrogen Novex Sharp Pre-stained Marker, band sizes indicated in kDa. Arrows indicate bands 1, 2 and 3 cut out for peptide mass fingerprinting, as described in text.

To identify the component of the supernatant responsible for biofilm dispersal activity, the supernatant of strain *B. licheniformis* EI-34-6 (grown for 7 days in an AMS bioreactor) was subjected to bioassay guided fractionation. We tested multiple methods of concentrating the supernatant (rotary evaporation, freeze drying, TCA precipitation) and multiple gel filtration media (Sephadex™ G-50, Sephadex™ LH-20, Superose™ 12, GE Healthcare, UK). The best separation of the active fraction was achieved using TCA precipitation followed by fractionation using Superose™ 12 gel filtration. Proteins in the active fraction were concentrated by TCA precipitation again and analysed by SDS page giving three distinct protein bands (Fig. 3).

### Identification of proteins in the active fraction by peptide mass fingerprinting

Proteins in the active fraction were identified by SDS-page and peptide mass fingerprinting. The lowest molecular weight band on the SDS-page gel, approx 12 kDa, contained two small proteins, both of them nucleases. The most abundant protein was Barnase (locus_tag: BL03601), a secreted ribonuclease, and the other protein was NucB (locus_tag: BL00126), a secreted deoxyribonuclease. The second band, cut out at approximately 30 kDa, contained three different proteins. The most abundant protein was protein YckK (locus_tag: BL01829) from the solute-binding family. Also present was the glycine betaine ABC transporter (opuAC; locus_tag: “BL01556”) and the ribonuclease present in the 12 kDa band. The third band, cut out at approximately 36 kDa, contained three different proteins. The most abundant protein was the same glycine betaine ABC transporter found in the 30 kDa band. Also present was an ABC transport system substrate-binding protein and probably also the putative extracellular solute-binding protein YckB (locus_tag: BL01818). We tested the correlation between biofilm dispersal activity and DNase activity and found that all culture media and fractions capable of dispersing biofilms also contained DNase activity (data not shown). Based on these results the two most likely candidates to have biofilm dispersal activity, the predicted ribonuclease Barnase and the predicted deoxyribonuclease NucB, were cloned.

### Overexpression of NucB and Barnase

**Figure 4:**
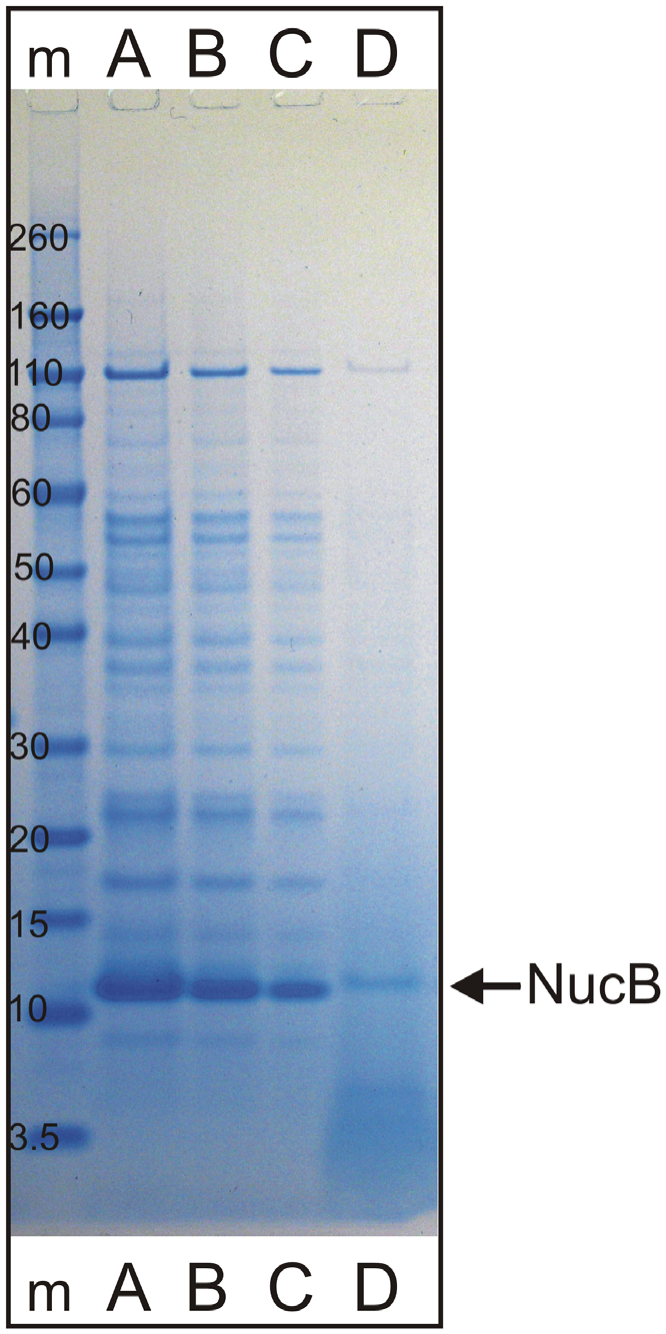
Heterologous overexpression of NucB in *B. subtilis* NZ8900. Lane **m** = Invitrogen Novex Sharp Pre-stained Marker, band sizes indicated in kDa. Lanes **A–C**: 20 fold concentrated TCA precipitated supernatant of strain *B. subtilis* NZ8900+pNZ8901-*nucB*, loaded 20 µl (**A**), 10 µl (**B**), 5 µl (**C**). Lane **D**: loaded 20 µl unprocessed supernatant. Arrow indicates NucB position.

Cloning and overexpression of NucB and Barnase was performed in *Bacillus subtilis* NZ8900. Primers were designed to amplify both identified nuclease genes based on the published genome sequence of *B. licheniformis* DSM13 [16]. Both genes were successfully amplified from *B. licheniformis* EI-34-6 chromosomal DNA and cloned into the SURE expression vectors pNZ8901 and pNZ8902 using *E. coli* as an intermediate host. Both vectors were transformed to the SURE expression strain *B. subtilis* NZ8900 [17]. Direct over-expression of NucB was successful with expression being under the control of pSpaS induced by subtilin (Fig. 4), whilst over-expression of Barnase required the inclusion of the gene downstream of Barnase, Barstar. Barstar is known to inhibit the intracellular RNase activity of the pre-barnase [19], thus allowing the over-expression and secretion of the Barnase itself, although on a lower level than for NucB.

**Figure 5:**
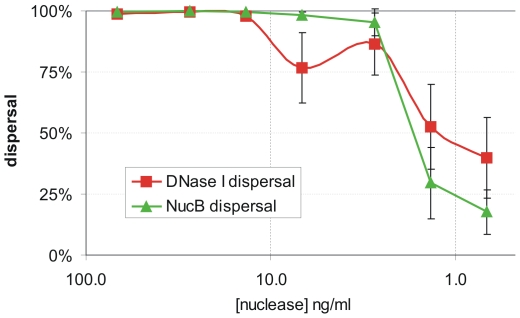
Comparison between NucB and DNaseI mediated biofilm dispersal. Efficiency of dispersal of 24 hour old *B. licheniformis* DSM13 biofilms by the tested nucleases in decreasing concentrations. Dispersal of the target biofilm was determined using a 96 well microtitre plate setup, using a concentration range of either *B. subtilis* supernatant containing NucB or commercially available DNaseI. For every data point, the average of at least 6 independent wells was taken, and the experiment was repeated three times.

The supernatant of *B. subtilis* NZ8900 containing the induced overexpression construct of NucB had strong DNase activity (data not shown) and was capable of dispersing established biofilms, whereas a control with an induced empty vector was not. The non-purified supernatant could disperse biofilms at NucB concentrations as low as 3 ng/ml (Fig. 5).

Heterologously expressed Barnase was also tested, and compared to commercially available RNaseI in its ability to disperse biofilms. No dispersal was observed using either heterologously produced Barnase or RNaseI in concentrations up to 10% supernatant/well or 200 units/ml respectively. We also tested the activity of Barnase in combination with bovine DNaseI or NucB, but no significant increase in dispersal was found compared to NucB or DNaseI alone (data not shown).

The *B. licheniformis* EI-34-6 genes for NucB and Barnase were sequenced (MWG operon, UK) to identify potential differences with the known sequenced strain. Both *nucB* (22bp of 428bp = 5.1%) and barnase (21bp of 455bp = 4.6%) contained base pair substitutions, leading to 4 amino acid changes in the NucB protein and 6 amino acids changes in the Barnase protein, compared to the DSM13 sequence. The sequences are available through Genbank, accession numbers HQ112343 (*nucB*) and HQ112344 (*barnase*).

### Comparison of NucB and Bovine DNase I biofilm dispersal ability

Bovine DNaseI gave full dispersal of the bacterial biofilm above 15 ng/ml. At concentrations below 15 ng/ml there was still partial dispersal, and at the lowest level tested (0.7 ng/ml) ∼35% of the biofilm was dispersed (Fig. 5). For NucB, above 3 ng/ml there was full biofilm dispersal whilst below this concentration the dispersal activity dropped rapidly, and at 0.7 ng/ml only 15% of the biofilm was dispersed. From this experiment it is clear that eukaryotic DNaseI and bacterial NucB have a very different dose response curve in relation to biofilm dispersal. Importantly, NucB is fully dispersing the biofilm at a w/v concentration 5 times lower than that of DNaseI. (Fig. 5) The defined cut off of activity with NucB, compared to the more gradual loss of activity of DNaseI, suggests that the bacterial nuclease is better adapted to disrupt eDNA present in bacterial biofilms.

## Discussion

It has been comprehensively demonstrated that DNA is present in biofilms [8], [9] and that it plays an important structural role in biofilm architecture [11], [13], [20], [21]. It has also been shown that some microbial species have significant quantities of RNA present within the biofilm matrix [22]. Furthermore, it has been observed that some bacteria, such as *P. aeruginosa*, have developed regulatory circuits that can utilize the eDNA present in the biofilm as a nutrient source during phosphate starvation through expression of extracellular nucleases [23]. In addition, based on the presence of eDNA in biofilms, commercially available bovine or human DNaseI has been used to treat bacterial biofilm infections [9], [11], [21].

Here we report that bacteria appear to be able to actively employ endogenously-derived nucleases in order to influence the biofilm in which they naturally grow. Bacteria have evolved a neat solution to escape their own biofilms and appear to use the same approach to disperse biofilms of competing species in a controlled and precise manner. Uniquely, we set out to identify the observed dispersal activity of a bacterial culture supernatant against competing species [24]. As a result of this, we have shown that bacteria use secreted nucleases as an elegant strategy to prevent *de novo* biofilm formation and that these nucleases can also to be used to disperse established biofilms of both Gram positive and Gram negative bacteria.

We observed that during biofilm growth *B. licheniformis* secretes both a ribonuclease and a deoxyribonuclease. We demonstrated that the deoxyribonuclease NucB is sufficient for dispersal of several target biofilms. Despite the observation of a ribonuclease (Barnase) within active supernatant we did not observe an additional effect of the ribonuclease on dispersal efficacy *in vitro*. However, it is tempting to speculate that *in vivo*, the Barnase does have an important role. As already observed by Catlin in 1954, the addition of RNase could improve the efficiency of the DNase in degrading eDNA, which lead him to conclude some biofilms may contain a DNase inhibitor and that “this DNase inhibitor is a ribonucleic acid” [8]. Although not observed our experimental setup, a possible reason for the expression of Barnase and NucB together could be found in the inhibitory effect of RNA on DNase activity.

In *Bacillus subtilis*, a close relative of *B. licheniformis*, NucB expression was studied in detail and found to be controlled by a sporulation specific promoter [25]. We suggest that the DNase activity of NucB leads to biofilm dispersal or permeabilization during sporulation, allowing spores to more readily disperse from the biofilm into the wider environment. It is also tempting to speculate that *B. licheniformis* uses extracellular DNases in order to disrupt biofilms of competing bacteria as a method of competing for resources. Combined with the expression of bacitracin during the same mode of growth *B. licheniformis* appears to use an elegant multi-approach strategy against competing species, breaking up their biofilms and secreting antibiotics at the same time.

The viscoelastic and adhesion properties of biofilms have been examined, however to date, work has mainly focused on the influence of polysaccharides on the physical properties of the matrix [26], [27]. Linear high molecular weight DNA is known to lead to an increase in viscosity of aqueous solutions [28], thus eDNA is likely to contribute to the viscoelastic and adhesion properties of the biofilm matrix. The release of a nuclease would therefore represent an elegant solution which might allows cells to escape from a “sticky” eDNA matrix such as those present in biofilms. The use of DNA to “trap” bacterial cells is also observed in the eukaryotic immune response, where lysis of neutrophils can create neutrophil extracellular traps, or NETs, which contain large amounts of eDNA. These are thought to play an important role in capturing invasive pathogens [29]. The existence, therefore of nuclease activity may also allow bacteria to escape from such traps.

Our observations are also supported by work with *Staphylococcus aureus*. The amount of eDNA released into the biofilm matrix and the activity of secreted *Staphylococcal* nucleases also influences biofilm structure [13]. Thus, the use of eDNA and nuclease to control of biofilm architecture does seem to be a strategy adopted by several groups of bacteria.

It is increasingly apparent that DNA is used both by bacteria and eukaryotes as a structurally important adhesin. We show here that the release of a matching nuclease represents an effective anti-adhesin strategy, and the combination of both mechanisms brings about a very elegant method allowing considerable fine-tuning of the system. It is clear that the function of DNA goes beyond its role as a carrier of genetic information alone but, in the form of eDNA, is a key component to control the dynamic building, reshaping and destruction of microbial biofilms.
